# Telemonitoring of Home-Based Biking Exercise: Assessment of Wireless Interfaces

**DOI:** 10.2196/41782

**Published:** 2022-10-12

**Authors:** Aref Smiley, Te-Yi Tsai, Wanting Cui, Irena Parvanova, Jinyan Lyu, Elena Zakashansky, Taulant Xhakli, Hu Cui, Joseph Finkelstein

**Affiliations:** 1 Center for Biomedical and Population Health Informatics Icahn School of Medicine at Mount Sinai New York, NY United States

**Keywords:** telerehabilitation, wireless interface, remote cycling, home-based exercise

## Abstract

**Background:**

Telerehabiliation has been shown to have great potential in expanding access to rehabilitation services, enhancing patients’ quality of life, and improving clinical outcomes. Stationary biking exercise can serve as an effective aerobic component of home-based physical rehabilitation programs. Remote monitoring of biking exercise provides necessary safeguards to ensure exercise adherence and safety in patients' homes. The scalability of the current remote monitoring of biking exercise solutions is impeded by the high cost that limits patient access to these services, especially among older adults with chronic health conditions.

**Objective:**

The aim of this project was to design and test two low-cost wireless interfaces for the telemonitoring of home-based biking exercise.

**Methods:**

We designed an interactive biking system (iBikE) that comprises a tablet PC and a low-cost bike. Two wireless interfaces to monitor the revolutions per minute (RPM) were built and tested. The first version of the iBikE system uses Bluetooth Low Energy (BLE) to send information from the iBikE to the PC tablet, and the second version uses a Wi-Fi network for communication. Both systems provide patients and their clinical teams the capability to monitor exercise progress in real time using a simple graphical representation. The bike can be used for upper or lower limb rehabilitation. We developed two tablet applications with the same graphical user interfaces between the application and the bike sensors but with different communication protocols (BLE and Wi-Fi). For testing purposes, healthy adults were asked to use an arm bike for three separate subsessions (1 minute each at a slow, medium, and fast pace) with a 1-minute resting gap. While collecting speed values from the iBikE application, we used a tachometer to continuously measure the speed of the bikes during each subsession. Collected data were later used to assess the accuracy of the measured data from the iBikE system.

**Results:**

Collected RPM data in each subsession (slow, medium, and fast) from the iBikE and tachometer were further divided into 4 categories, including RPM in every 10-second bin (6 bins), RPM in every 20-second bin (3 bins), RPM in every 30-second bin (2 bins), and RPM in each 1-minute subsession (60 seconds, 1 bin). For each bin, the mean difference (iBikE and tachometer) was then calculated and averaged for all bins in each subsession. We saw a decreasing trend in the mean RPM difference from the 10-second to the 1-minute measurement. For the 10-second measurements during the slow and fast cycling, the mean discrepancy between the wireless interface and tachometer was 0.67 (SD 0.24) and 1.22 (SD 0.67) for the BLE iBike, and 0.66 (SD 0.48) and 0.87 (SD 0.91) for the Wi-Fi iBike system, respectively. For the 1-minute measurements during the slow and fast cycling, the mean discrepancy between the wireless interface and tachometer was 0.32 (SD 0.26) and 0.66 (SD 0.83) for the BLE iBike, and 0.21 (SD 0.21) and 0.47 (SD 0.52) for the Wi-Fi iBike system, respectively.

**Conclusions:**

We concluded that a low-cost wireless interface provides the necessary accuracy for the telemonitoring of home-based biking exercise.

## Introduction

Telerehabilitation overcomes the barriers of distance and time using telecommunications and enables remote delivery of health care services from clinicians to patients’ homes. Advances in telerehabilitation technology has brought about a possibility of a remotely supervised rehabilitation program through real-time communication, tracking of physical activities of a patient, monitoring vital body functions, and rehab physical therapy [1-3]. Telemedicine use has grown significantly during the COVID-19 pandemic [4] when the World Health Organization encouraged physical distancing [5]. Telehealth approaches may be instrumental for supporting home-based cycling exercises in people with chronic health conditions especially when safety and adherence with exercise prescription can be monitored in real time [6]. Existing solutions are primarily designed for healthy athletes and are characterized by high cost and lack of functionality that allows health professionals to monitor cycling exercise and provide feedback to patients in a timely fashion. Limited research has been conducted on low-cost wireless interfaces for the real-time remote monitoring of cycling exercise in a telerehabilitation setting that promotes upper and lower limb rehabilitation.

Cycling exercise equipment is often used to facilitate training of the upper and lower extremities, and is widely available in rehabilitation facilities that can oversee patient exercise [7]. Cycling exercise training was shown to improve clinical outcomes in patients in hemodialysis [8], patients in recovery stage after hip fracture [9], patients with mechanical ventilation [10], patients with acute recovery stage after stroke [11], patients with chronic pulmonary disease [12], patients with chronic health conditions [13], patients with Parkinson disease [14], hemiparetic patients [15], patients with COVID-19 [16], and in-bed critically ill patients [17]. Patients’ engagement in telerehabilitation could be promoted using gaming and consumer appliances [18-22], where patients get motivation to engage in enjoyable play behavior that involves useful therapy-related activities [23].

Cycling exercise equipment is widely available at homes at low cost; however, lack of remote connectivity with a team of rehabilitation professionals to monitor exercise progress in real time makes it far from being effective in practice. Providing simple real-time visualizations and numerical expressions in addition to designing an alert system, preventing exertion levels exceeding those approved by a rehabilitation team, would highly improve the remote exercise effectiveness and safety. We previously showed a high level of system acceptance by the study participants for the initial iBikE design system [24]. The data obtained from this study provided the basis for the development and testing of optimal customized telerehabilitation programs.

The goal of this study was to design and test the accuracy of two low-cost wireless interfaces for the telemonitoring of home-based cycling exercise systems. The major difference of the two systems was the communication protocol. The first system used Bluetooth Low Energy (BLE) to communicate between the iBikE and tablet PC. The second system used Wi-Fi to communicate between the iBikE and tablet PC.

## Methods

### Overview

We developed two iBikE systems that use either Wi-Fi or BLE protocol to communicate between the tablet PC and iBikE. To evaluate and assess the accuracy and functionality of the systems, we tested both systems in two separate experiments. During each experiment, we used a laser tachometer to measure the bike speed. A total of 9 healthy individuals were asked to hand cycle in each experiment. Finally, collected data from the systems were compared to their representative collected data from the laser tachometer.

**Figure 1:**
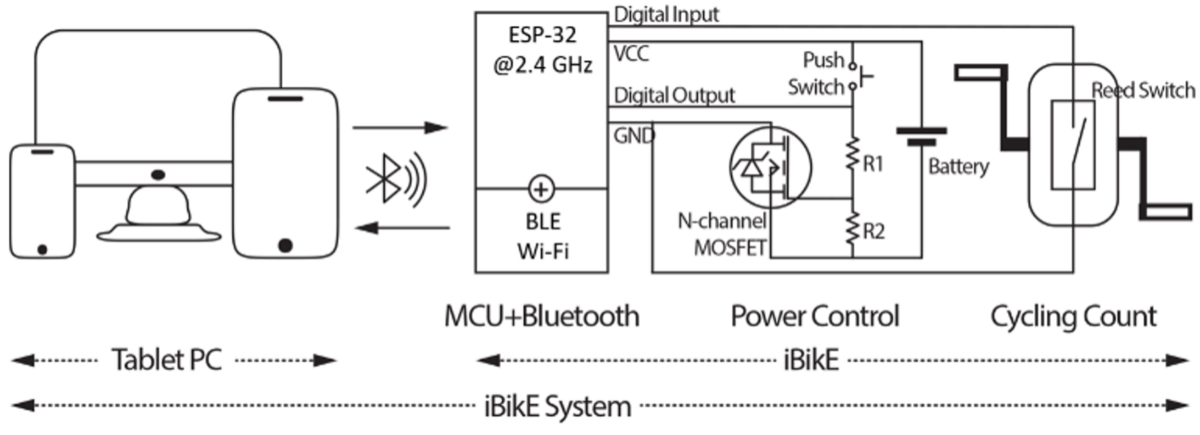
System design. Both developed systems have the same hardware and user interface. The major difference between the two systems is the communication protocol. They communicate through either Wi-Fi or BLE. BLE: Bluetooth Low Energy; MCU: microcontroller unit; VCC: Voltage Common Collector; GND: ground; MOSFET: metal–oxide semiconductor field-effect transistor.

We used the same hardware and user interface for both designs. The iBikE system has two main parts: (1) a tablet PC application and (2) an iBikE (Figure 1). Recorded data were sent through wireless communications from built-in systems in the iBikE to the PC tablet. In addition, data were provided to telerehabilitation users when they were engaged in cycling exercises. To make it easy to use, the iBikE itself had only one physical button and the tablet PC had a touch screen button to start/stop the exercise session.

### Tablet PC Application

**Figure 2:**
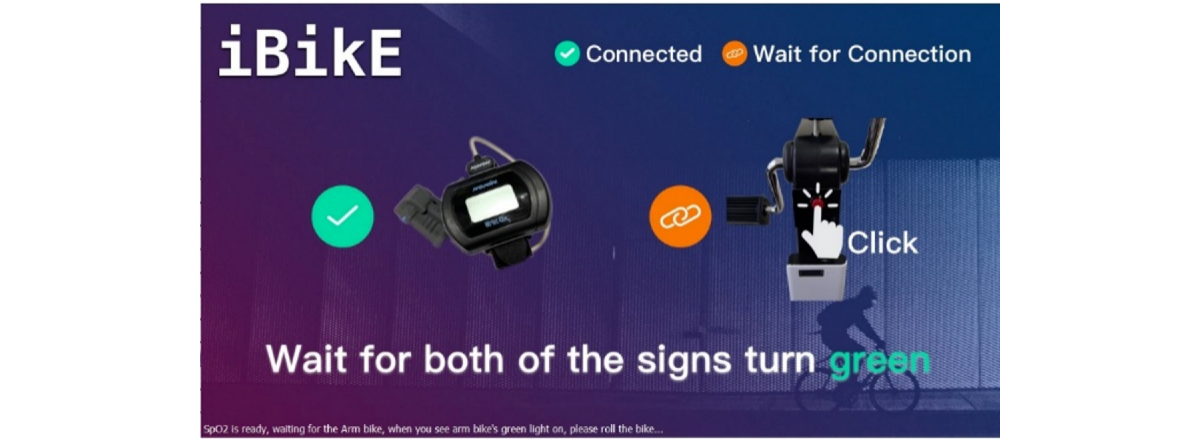
User interface. User needed to push a button to pair the application to the oximeter. They also needed to push the button on the right to pair the application to the iBikE equipment to start the cycling session.

**Figure 3:**
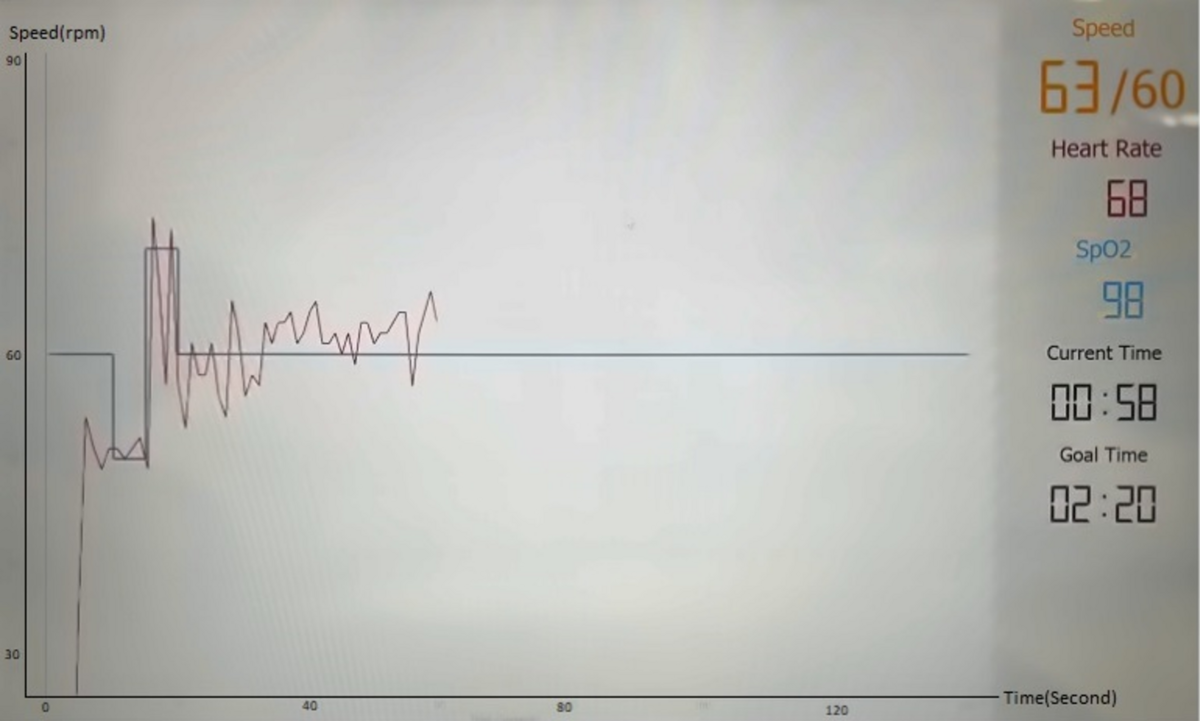
Real-time monitoring of the speed (rpm) versus time (second) during cycling. Information taken from the oximeter, including pulse rate and oxygen saturation, were also displayed during the cycling sessions. The image was taken with a camera and filtered for better illustration. rpm: revolutions per minute.

We developed a touchscreen-operated application on a tablet PC using Universal Windows Platform, which can be run on any Windows 10 operating system. We used C# programming language to develop the application. Two buttons were designed in the first page of the application, Pulse Oximeter and iBikE buttons. To display oxygen saturation and pulse rate data while the user was cycling, the user needed to push the Pulse Oximeter button to connect the wrist-worn pulse oximeter. We used the WristOx2, model 3150 [25], which connects to the tablet PC via BLE. By pressing the Pulse Oximeter image, the application first scans and then pairs the pulse oximeter through the BLE to the tablet PC. This turns its representative device button on the application to green (Figure 2). The application uses a standard universally unique identifier (UUID) to get the characteristics and their values. Successful pairing of the microcontroller unit (MCU) of the wireless interface with the tablet PC allows the user to start an exercise session and to track biking progress in real time. In addition, it allows the received data from the pulse oximeter to be displayed on the exercise page (Figure 3). Before starting the cycling session, the user had to activate the pulse oximeter by clicking on its image in the first page of the application. To start the cycling session, users needed to first push the physical button on the bike to turn on the iBikE equipment. Next, they needed to connect the tablet to the iBikE equipment by pressing its representative button on the application (Figure 2). This resulted in the connecting of the application to the iBikE through either Bluetooth or Wi-Fi and took the user to the second page of the application (Figure 3). On the second page, by starting the exercise, the real-time exercise feedback interface via the tablet PC was activated, and the user could monitor the real-time speed in revolutions per minute (RPM). In addition, the real-time pulse rate and oxygen saturation data were sent from the oximeter to the tablet and could be monitored on the second page. The user’s entire exercise data was stored on the local server and in .csv files.

### iBikE Equipment

There were three main modules in the iBikE exercise equipment (Figure 1): (1) control and computing module, (2) magnetic switch module (reed sensor), and (3) either Bluetooth or Wi-Fi communication module.

#### Control and Computing Module

In this project, we used FireBeetle ESP-32, which is a low-power consumption MCU designed for Internet of Things projects. It integrated a Dual-Core ESP-WROOM-32 module, which supports MCU and Wi-Fi and Bluetooth dual-mode communication. It also supports a 3.7V external lithium battery power supply. The iBikE first read the information provided by the reed switch sensor through the ESP32 MCU to obtain relevant cycle information and then sent the data to the tablet PC through either Bluetooth or Wi-Fi. Real-time cycling information, the intervals (milliseconds), was measured through the calculation algorithm in the MCU. The intervals were then delivered to the tablet PC via either the Wi-Fi or Bluetooth communication module. The reed switch was used to measure the time required to complete a single cycling of the iBikE.

#### Magnetic Switch Module

The reed sensor (magnetic switch) is a sensor that converts magnetic field changes into electrical signals. It consists of a pair of ferromagnetic flexible metal contacts, normally open, in a sealed glass envelope. By holding the magnet to the magnetic switch of the iBikE equipment, the magnetic switch detects the change in the magnetic field and closes the metal contacts, resulting in the flow of an electrical signal. By removing the magnet from the magnetic field, the magnetic switch detects the change and stops the flow of the electrical signal. In the iBikE equipment, every rotation of the pedals (one cycle) was detected by the magnetic switch. Continuous sampling of the electrical signal flow from the switch by the MCU provided cycling time intervals. The user’s departure from the iBikE was detected if there was no detection of on/off changes in the sampled electrical signal for more than 90 seconds.

#### Bluetooth or Wi-Fi Communication Module

We developed two separate iBikE systems to transmit cycling intervals from the iBikE system to the PC tablet using either Wi-Fi or BLE communication protocol in each system. In BLE communication mode, the PC tablet was directly connected to the MCU integrated inside the iBikE equipment. Cycle intervals were first converted into two bytes: low byte (the least significant part of an integer) and high byte (the most significant part of an integer). The byte packets were then sent to the tablet PC. We defined our custom UUID to transfer values from the MCU to the tablet PC. In Wi-Fi communication mode, both the PC tablet and the MCU were connected to the same Wi-Fi network. User Datagram Protocol was used to send cycling intervals from the bike, configured as client, to the PC tablet, configured as server.

### Data Collection

Both developed iBikE systems had the same operating procedure. To start the cycling session, the user was required to first press the physical red button on the iBikE exercise equipment to wake up the MCU and to activate either the Wi-Fi or Bluetooth communication with the tablet PC. The user then needed to press the button on the first page of the application (Figure 2) to indicate the beginning of the exercise. This allowed the tablet PC to connect to the Wi-Fi/Bluetooth communication module and pair with the iBikE exercise equipment. After pairing the tablet PC to the iBikE, the prescribed cycling speeds appeared on the second page of the interface (Figure 3), where the user could exercise via the iBikE exercise equipment and receive feedback on the user’s exercise.

**Figure 4:**
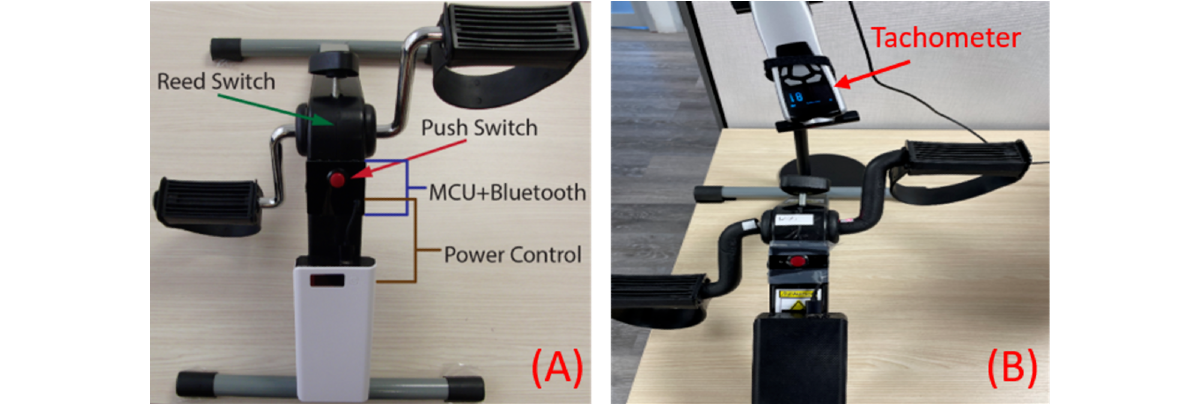
(A) Exercise equipment. The user interface is only a physical button to make it easy to use the equipment. All the components, including the reed switch, push switch, MCU and Bluetooth Low Energy modules, and power control module are inside the iBikE equipment. (B) We used a laser tachometer, DT-2100, Nidec-SHIMPO [15], in noncontact continuous measurement mode to detect the measured revolutions per minute in real time to later compare the results with data taken from the iBikE equipment. MCU: microcontroller unit.

The accuracy of each iBikE system was checked using a laser tachometer, DT-2100, Nidec-SHIMPO [26]. The tachometer was used in noncontact continuous measurement mode to detect the measured RPM in real time (Figure 4B). The tachometer samples the continuous RPM measurements with 10 Hz sampling frequency. Collected data could be visualized in its PC software in real time. At the end of each session, recorded data could be saved in an Excel (Microsoft Corporation) file. Finally, the saved data during each cycling experiment were compared to their representative collected data from the iBikE system.

A single group of 9 individuals performed 3 sessions of hand cycling for each iBikE system, with a 1-minute duration for each session. They started with 1 minute of hand cycling at a slow pace, followed by 1 minute of rest. They then started with 1 minute of hand cycling at a medium pace, followed by 1 minute of rest. Finally, they started with 1 minute of hand cycling at a fast pace. The meaning of slow, medium, and fast was based on the user interpretation. Participants in the tests were 9 healthy individuals, aged between 21 and 37 years. They performed the tests on 2 different days. Data were collected from the iBikE system with the BLE communication mode on the first day, and data were collected from the iBikE system with Wi-Fi communication mode on the second day. Users completed a total of 18 sessions (54 phases). Data were collected from both the iBikE system and tachometer in parallel during each session. The iBikE system recorded completed cycling intervals and sent the data to the PC tablet. At the same time, the tachometer collected RPM values and sent them to a PC with a sampling rate of 10 Hz.

In addition to cycling information, which was sent by the MCU to the application, other information, including heart rate, SpO2 (oxygen saturation level), and Personal Activity Intelligence [27], was sent from the pulse oximeter to the application.

### Ethical Considerations

As there was no risk and the participants were all authors of this paper, we did not require institutional review board approval. Data were collected between May 2022 and June 2022. No protected health information were collected, and the resulting analytical data set was fully deidentified. No compensation was provided to the study participants.

## Results

**Figure 5:**
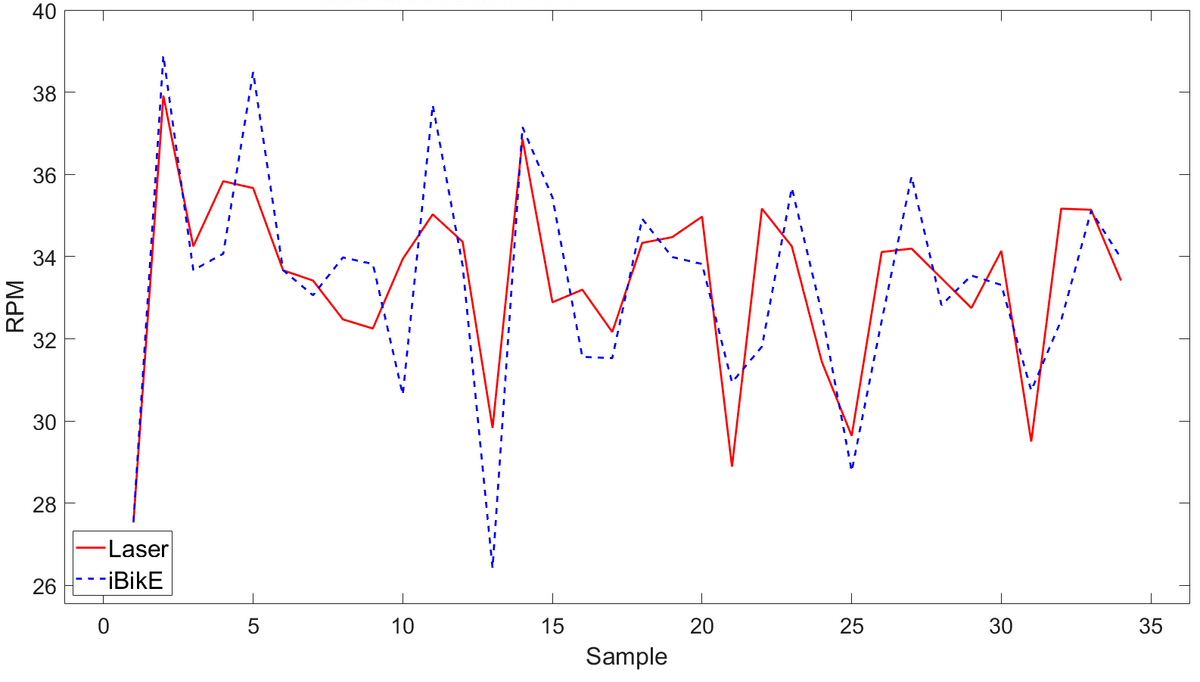
Measured RPM from the iBikE system versus the tachometer in a 1-minute session at a slow pace. RPM: revolutions per minute.

**Figure 6:**
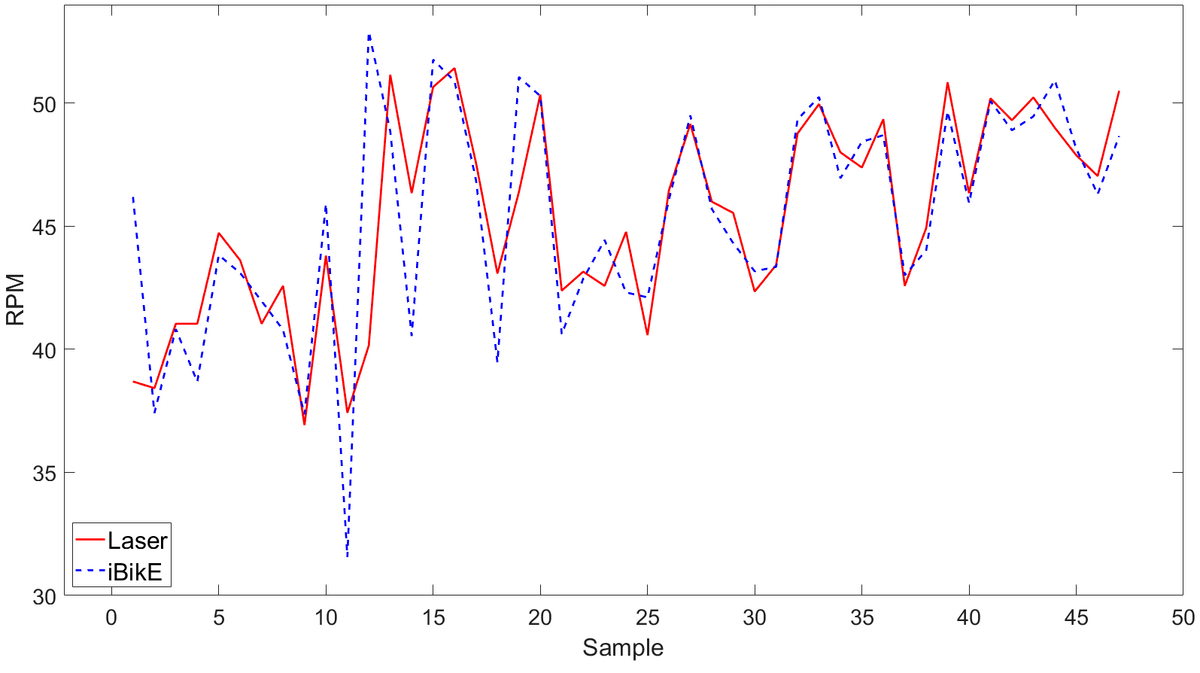
Measured RPM from the iBikE system versus a tachometer in 1-minute sessions at a medium pace. RPM: revolutions per minute.

**Figure 7:**
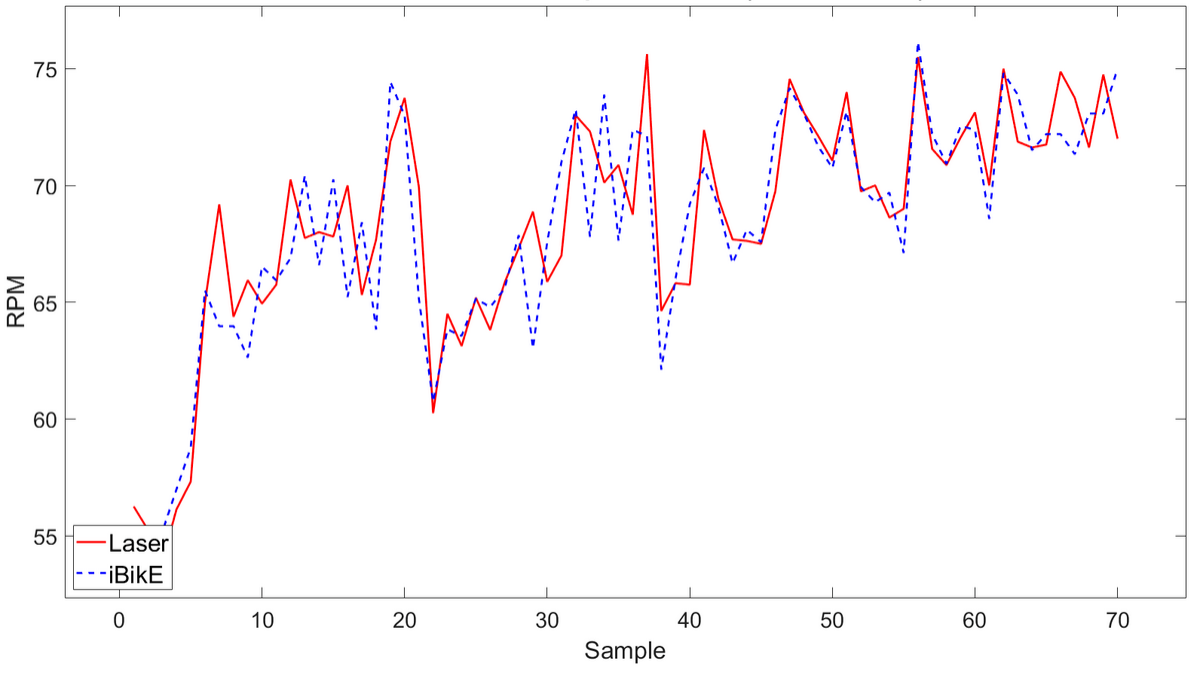
Measured RPM from the iBikE system versus a tachometer in 1-minute sessions at a fast pace. RPM: revolutions per minute.

The iBikE system accuracy was evaluated by comparing the collected data from the iBikE and its representative collected data from the tachometer for each session. We developed an algorithm using MATLAB R2022a (MathWorks) to compare the two collected data sets. The first step in the algorithm was to shift the collected data from the iBikE to match its representative collected data from the tachometer. This was done for all 54 (1 minute) collected data at slow, medium, and fast pace (Figures 5-7). All the figures belonged to the same individual, cycling with various paces in three sessions in 1-minute durations.

In the collected data at a slow pace, we had less samples collected from the iBikE compared to the medium- and fast-paced sessions for the same individual. This is because the iBikE sends the intervals (in milliseconds) whenever each cycle is completed. In the slow-paced session, it takes longer for the user to complete a cycle, and therefore, less samples are collected by the iBikE system for each session with the same duration (60 seconds). However, in the tachometer, the data collection sampling rate is 10 Hz in continuous mode for all sessions. Therefore, we had 600 collected samples for each 60-second session. To compare the data from the iBikE and its representative collected data from the tachometer, data from the tachometer was averaged to be the same size to its representative collected data from the iBikE (Figures 5-7).

Shifting collected data from the iBikE and matching its representative collected data from the tachometer allowed us to determine the starting and ending points of each session. The second step in the algorithm was to divide each session (60 seconds) into 4 subsessions: 10 seconds, 20 seconds, 30 seconds, and 60 seconds for both collected data from the tachometer and the iBikE systems. In a 10-second subsession, for example, both collected data from the iBikE and tachometer in each session (with 60-second durations) was divided into 6 bins of 10 seconds. The mean of the RPM were then calculated for every 10-second bin. The mean difference was then calculated for each bin (iBikE and its representative tachometer bin). Finally, the mean value (of the 6 bins) was calculated. Table 1 shows an example of the calculated mean for all subsections for one of the participants.

**Figure 8:**
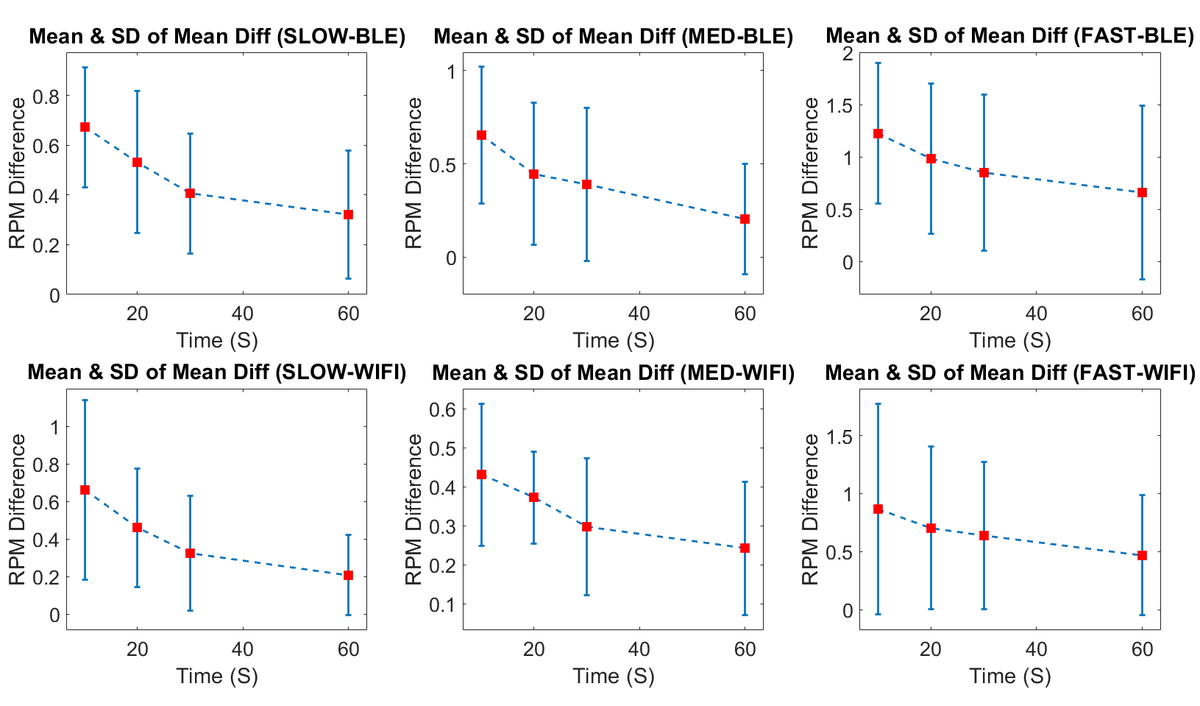
Means and SDs of the calculated mean differences in the 10-, 20-, 30-, and 60-second subsessions for all individuals for both the Wi-Fi and Bluetooth Low Energy iBikE systems. RPM: revolutions per minute.

Finally, the mean and SD of all the similar subsections for all participants were calculated and are shown in Tables 2 and 3 and Figure 8.

In each session, the mean and SD of the subsections showed a downtrend from 10-second measured means to 60-second ones. The trend was the same for all the sessions for both Wi-Fi and BLE systems. The maximum calculated means of subsections for each person’s collected data were 2.96 for Wi-Fi and 2.69 for BLE, both in 10 seconds of collected data at a fast pace. The minimum mean of the subsections for all participants’ collected data was 0.2 (SD 0.3) in the 60-second subsession at medium speed. For the Wi-Fi iBikE system, this number was 0.21 (SD 0.21) in the 1-minute subsession at slow speed.

**Table 1:** Example of calculated mean difference for each subsection (10 seconds, 20 seconds, 30 seconds, and 60 seconds) in each session (slow, medium, fast) for both the BLE and Wi-Fi iBikE systems for one of the participants. Each section is divided into 6 bins, 3 bins, 2 bins, and 1 bin. The difference of the revolutions per minute means is then calculated for each bin. The mean of the bins in each subsection is reported.

Subsections	BLE^a^ sections				Wi-Fi sections		
	Slow	Medium	Fast	Slow		Medium	Fast
10-s bins (6 bins), mean	0.69	0.87	0.95	0.53		0.53	0.7
20-s bins (3 bins), mean	0.38	1.14	0.7	0.27		0.5	0.37
30-s bins (2 bins), mean	0.42	1.13	0.52	0.13		0.1	0.39
60-s bin (1 bin), mean	0.17	0.1	0.08	0.15		0.07	0.15

^a^BLE: Bluetooth Low Energy.

**Table 2:** Mean and SD of all the collected differences between the wireless interface and tachometer in each subsection with similar bins for the Bluetooth Low Energy iBikE system.

Subsections	Slow pace sessions, mean (SD)	Medium pace sessions, mean (SD)	Fast pace sessions, mean (SD)
10-s bins	0.67 (0.24)	0.65 (0.37)	1.22 (0.67)
20-s bins	0.53 (0.29)	0.45 (0.38)	0.98 (0.72)
30-s bins	0.41 (0.24)	0.39 (0.41)	0.85 (0.75)
60-s bins	0.32 (0.26)	0.20 (0.30)	0.66 (0.83)

**Table 3:** Mean and SD of all the collected differences between the wireless interface and tachometer in each subsection with similar bins for the Wi-Fi iBikE system.

Subsections	Slow pace sessions, mean (SD)	Medium pace sessions, mean (SD)	Fast pace sessions, mean (SD)
10-s bins	0.66 (0.48)	0.43 (0.18)	0.87 (0.91)
20-s bins	0.46 (0.32)	0.37 (0.12)	0.70 (0.70)
30-s bins	0.32 (0.31)	0.30 (0.17)	0.64 (0.63)
60-s bins	0.21 (0.21)	0.24 (0.17)	0.47 (0.52)

## Discussion

### Principal Results

We designed and developed two systems that could be used as in-home exercise for patients requiring a telerehabilitation exercise system. We designed the system in a way that was easily accessible and required minimal experience from users to operate the system. Our new systems are low-cost and showed their capability and accuracy in communicating through either BLE or Wi-Fi. We evaluated our iBikE systems’ accuracy and functionality by comparing their recorded data with the collected data from the laser tachometer. The results showed that the minimum mean RPM difference was 0.2 (SD 0.3) for 1 minute of hand cycling at a medium-paced session for the BLE iBikE system. For the Wi-Fi iBikE system, the minimum mean RPM difference was 0.21 (SD 0.21) for 1 minute of hand cycling at a slow-paced session.

We made the user interface as simple as possible for the user to work with the iBikE system and to start/stop a session. By pushing a single physical button on the iBikE equipment (Figure 3), both systems easily connect to the PC tablet. This activated the BLE/Wi-Fi connection in the MCU and then waited for the user to push a button on the tablet PC screen to confirm the connection and to start the cycling session. The same button on the iBikE equipment could be used to stop the cycling session.

### Limitations

We used FireBeetle ESP-32, with integrated Dual-Core ESP-WROOM-32 module, which supports MCU and Wi-Fi and Bluetooth dual-mode communication. To accurately record the cycling intervals from the reed switch on falling edges, we used interrupt service routine (ISR) in our detection algorithm. Due to the capacitive structure of the reed switch [28] and external magnetic field effect [29], random falling edges (*false* interrupts) for every ~250 milliseconds were detected in the Wi-Fi iBikE system. For the BLE iBikE system, *false* interrupts happened every ~140 milliseconds. When the system battery got below its ~70% capacity, the effect of detecting *false* interrupts was more frequent and longer, ~180 milliseconds and ~300 milliseconds for the BLE and Wi-Fi systems, respectively. Therefore, the cycling interval detection algorithm used a hold off timer in the ISR to prevent the counter from incrementing on the *false* interrupts. This was one of the advantages of the BLE system over the Wi-Fi system, as the hold off timer was smaller (less than 200 ms) and, as a result, could detect faster RPM (up to 300) compared to the Wi-Fi system. In addition, the BLE iBikE system used less battery during similar sessions. The highest RPM value recorded during the fast-paced sessions was 142 RPM. Therefore, both systems could detect all the cycling intervals with no missing data. However, the issue should be considered for similar applications, where data need to be recorded faster with higher frequency.

To activate the Wi-Fi communication in the ESP-32 MCU, we needed to set the username and password of an active router nearby the iBikE to be connected to Wi-Fi and to communicate with the PC tablet. If the user moved to a new location with a new active router, the MCU embedded programing code needed to be updated with the new username and password. This requires an experienced person with special integrated development environment software (Arduino IDE, etc) to update the code with the new username and password. In addition, we needed to provide the IP address of the tablet PC in the programing code to communicate to the tablet PC. However, the BLE system did not have these limitations. Our suggestions for using the BLE iBikE system over the Wi-Fi system are limited to this developed system, our application’s needs, and the system’s data results. Both BLE and Wi-Fi technologies have their strong and weak sides. When the application requires a big data transfer with faster speed and with high security, for example, Wi-Fi is a better choice.

### Conclusions and Future Work

The iBikE system consisted of a sensor for sampling fast cycles, detecting algorithms for the patient’s exit from the program, programs for collecting sensor data and communicating with a tablet PC, user interfaces for displaying the iBikE and patient data and entering control variables, and follow-up records and data collection systems. A low-cost wireless interface provided the necessary accuracy for the telemonitoring of home-based biking exercises. This iBikE system is novel because it can capture and communicate real-time exercise cycling data for a rehabilitation team using a reliable low-cost wireless interface.

In previous work, we demonstrated high acceptance and positive impact of physical telerehabilitation in patients with chronic pulmonary conditions [30], multiple sclerosis [31], and geriatric syndromes [32]. The next step is to evaluate the impact of home-based cycling telerehabilitation programs that include real-time exercise monitoring and data-driven feedback from rehabilitation teams in a clinical trial setting, which includes a diverse spectrum of patients enrolled for a prolonged period of time. Short-term and long-term cycling effects should be investigated and compared to the existing rehabilitation outcomes implemented in outpatient clinics. Telerehabilitation programs will be assessed by their impact on aerobic fitness, clinical outcomes, health care use, and exercise adherence.

## Abbreviations

BLE: Bluetooth Low Energy
ISR: interrupt service routine
MCU: microcontroller unit
RPM: revolution per minute
UUID: universally unique identifier
